# Supplementary material for: Arpp19 Promotes Myc and Cip2a Expression and Associates with Patient Relapse in Acute Myeloid Leukemia
Source: Cancers (Basel). 2019 Nov 11;11(11):1774. doi: 10.3390/cancers11111774 (PMC6895887; doi:10.3390/cancers11111774)
Supplement: Supplementary file 1 [file cancers-11-01774-s001.zip › cancers-635461-supplementary figures.pdf]

Supplementary Materials: Arpp19 Promotes Myc and Cip2a Expression and Associates with Patient Relapse in Acute Myeloid Leukemia

Eleonora Mäkelä, Eliisa Löyttyniemi, Urpu Salmenniemi, Otto Kauko, Taru Varila, Veli Kairisto, Maija Itälä-Remes and Jukka Westermarck

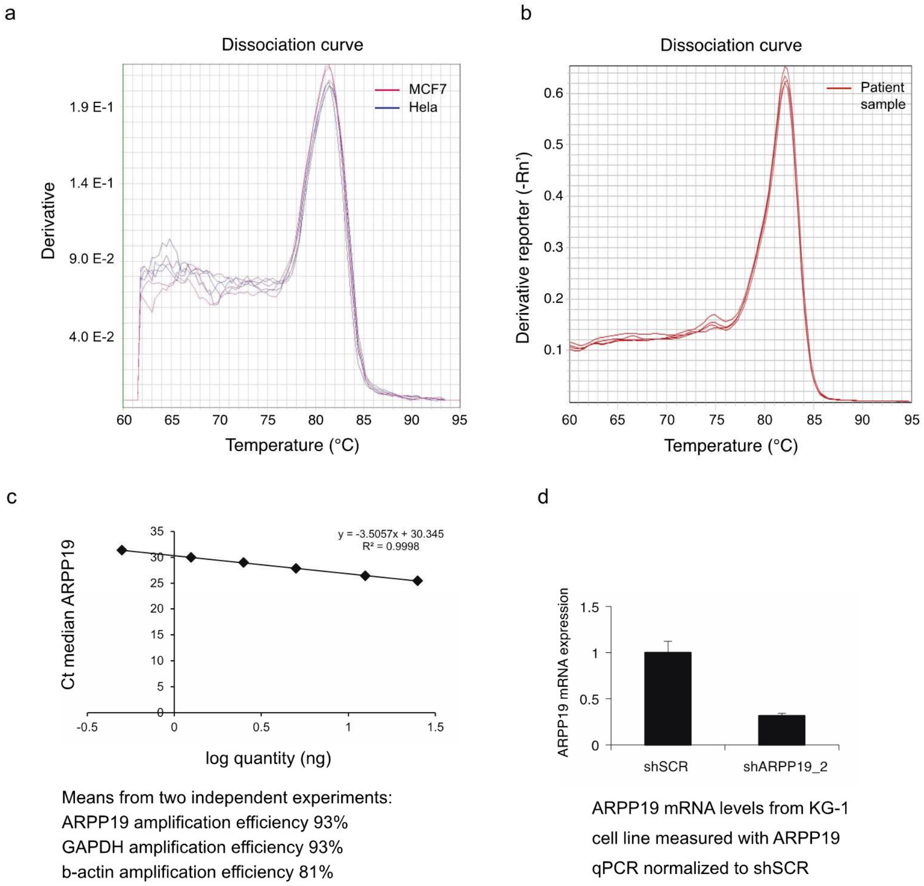

Figure S1 Validation of ARPP19 specific RQ-PCR. **a**) and **b**) Melting curves from RQ-PCR of ARPP19 gene with primers indicated in Supplemental table 7. Amplicons from both MCF7 and Hela cell line samples, as well as diagnosis phase AML patient sample, reveal a single peak following melting curve analysis. **c**) Amplification efficiency of ARPP19 RQ-PCR used in this study was 93% when measured with standard curve analysis. Shown is representative standard curve, but similar results were extracted from two independent experiments. Control genes used in the study, GAPDH and b-actin, showed amplification efficiencies of 93% and 81%, respectively. **d**) RQ-PCR specificity validation of ARPP19 assay on ARPP19 shRNA transduced KG1 cells.

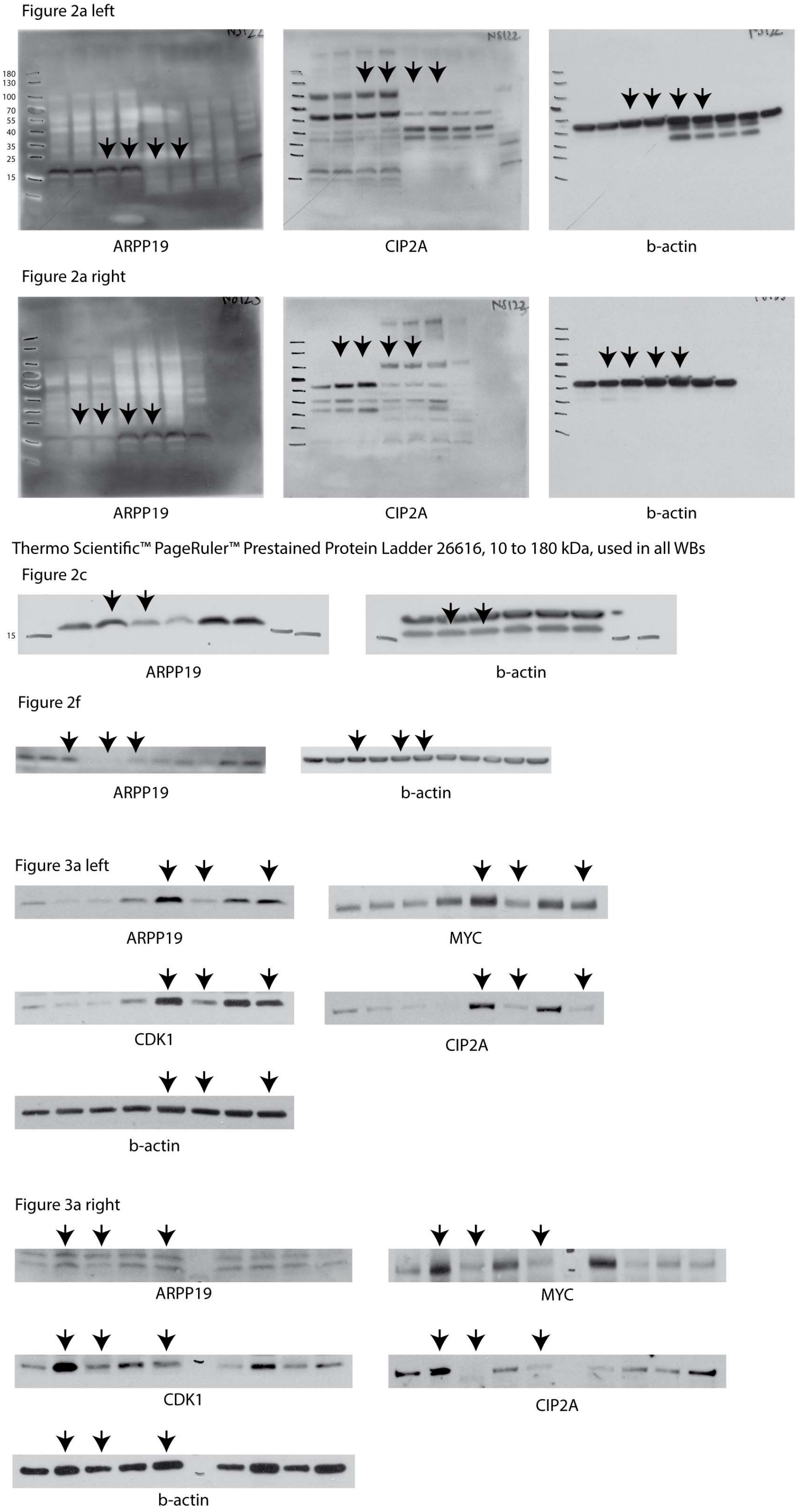

Figure S2. Original western blots for all main figures included in the article.

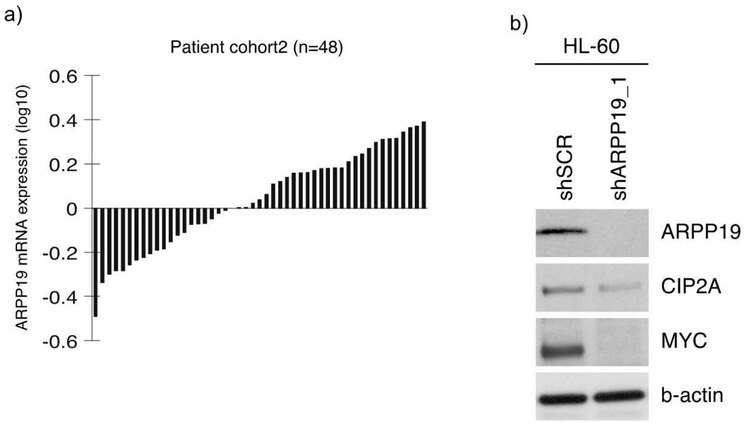

Figure S3 Validation of ARPP19 overexpression in AML cohort2. **a**) Waterfall blot of ARPP19 mRNA expression in patient cohort2 (n = 48) normalized to GAPDH & b-actin expression and a pooled normal bone marrow sample (n = 56). ARPP19 was 58% (n = 28) overexpressed in the sample panel. **b**) Representative western blot analysis of ARPP19, CIP2A and MYC expression in HL-60 cell line stably transduced with scrambled (shSCR) or ARPP19 shRNAs (shARPP19_1).

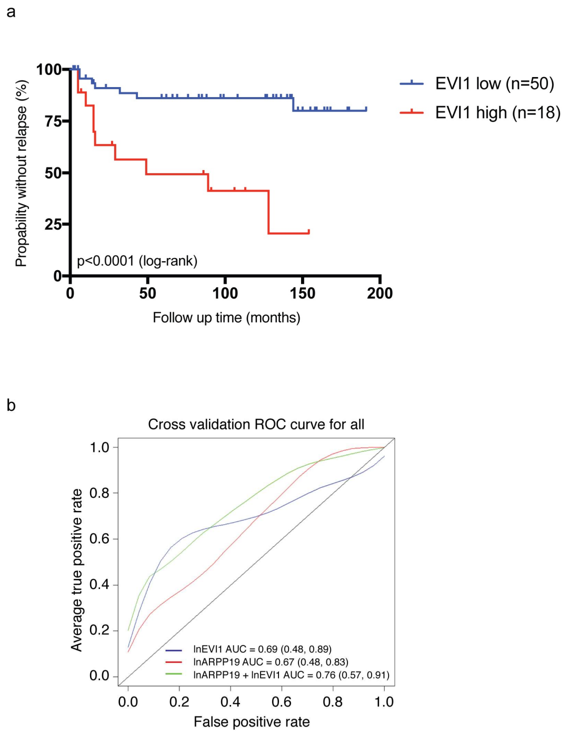

Figure S4. Kaplan-Meier curve for time to relapse according to EVI1 mRNA expression in cohort1. **a**) High EVI1 expression is significantly associated with shorter relapse free time of AML patients, $p < 0.0001$ by log-rank test. **b**) The ROC curve analysis on the abilities of ARPP19 and EVI1 gene expression at diagnosis in prediction of relapse in AML patients. AUC values and 95% confidence intervals are shown for each analysis.

© 2019 by the author. Licensee MDPI, Basel, Switzerland. This article is an open access article distributed under the terms and conditions of the Creative Commons Attribution (CC BY) license (http://creativecommons.org/licenses/by/4.0/).
